# Concurrent Tumors Revealed by an Autopsy-A Case Report and Literature Review

**DOI:** 10.1155/2022/2308065

**Published:** 2022-06-01

**Authors:** Miruna Cristian, Mădălina Boșoteanu, Mariana Așchie, Angelica Potamian, Cătălin Adrian Boșoteanu, Gabriela Izabela Bălțătescu

**Affiliations:** ^1^Department of Clinical Pathology, “St. Apostle Andrew” Emergency County Hospital, Constanta, Romania; ^2^Center for Research and Development of the Morphological and Genetic Studies of Malignant Pathology-CEDMOG, “Ovidius” University of Constanta, Constanța, Romania; ^3^Faculty of Medicine, “Ovidius” University of Constanta, Constanța, Romania; ^4^Academy of Medical Sciences, Bucharest, Romania; ^5^Department of Radiology, Clinical Pneumophthisiology Hospital, Constanta, Romania

## Abstract

**Introduction:**

Multiple primary malignant neoplasms are an uncommon phenomenon, given the very low incidence of two or more different tumors, while neoplasm may be limited to a single organ or may involve multiple separate anatomical organs. The main purpose of this study is to highlight the importance of morphological and immunohistochemical tests to distinguish the origin of the primary tumor. *Case Presentation*. We report the case of a 65-year-old deceased male, presenting multiple tumors in the lung, stomach, kidneys, and adrenal organs. The main symptoms presented by the patient were dyspnea with a range of 77% with oxygen saturation, fatigability, and productive cough. Histopathological examination revealed a solid and papillary lung adenocarcinoma, concurrent with tubular gastric adenocarcinoma. Immunohistochemical testing was mandatory by using a panel of seven monoclonal mouse antibodies (TTF-1, Napsin A, CK7, CK20, p40, synaptophysin, and chromogranin A). The pulmonary tumoral immunophenotype (positive for TTF-1, Napsin A, CK7; negative for CK20, p40, synaptophysin, and chromogranin A) confirms the diagnosis of primary lung ADC and invalidates the hypothesis of a metastasis arisen from a gastric adenocarcinoma or other forms of lung cancer.

**Conclusion:**

The importance of the ancillary test is to distinguish a primary tumor from a metastatic one.

## 1. Introduction

Multiple primary malignant neoplasms (MPMN) are an uncommon phenomenon as their incidence scarcely reaches 0.99% for two or more different tumors [1]. The criteria for multiple malignant tumors, defined in 1932 by Warren and Gates [2], is that each tumor must present a form of malignancy which clearly differentiates from the other tumors, while the possibility of one of them being a metastasis from the other must be excluded [3]. Neoplasms may either be limited to a single organ or involve multiple separate anatomical organs.

The North American Association of Central Cancer Registries (NAACCR) classifies MPMN into two categories: (1) synchronous, in which cancers occur at the same time (the Surveillance Epidemiology and End Results Programme (SEER) definition is within two months) [4] and (2) metachronous, in which the cancers follow in sequence, more than six months apart [4]. Synchronous MPMN, with lung and gastric cancer, is a rare event. In a retrospective study of Kurishima et al. (2009), 3.2% of 1391 patients with lung cancer (LC) had previous or simultaneous gastric cancer (GC) and smoking had been proven to be a risk factor, as the proportion of smokers was higher among lung cancer patients with GC than those without [5].

LC is one of the deadliest diseases worldwide with a 5-year survival rate of approximately 15% [6]. Globally, the overall lifetime risk of LC is about 1/13 for men and 1/16 for women [7]. There are two main forms of primary LC, classified by the type of cells in which the cancer starts to grow: non-small-cell lung cancer (NSCLC) and small-cell lung cancer (SCLC). NSCLC is the major subtype of LC, which can be further divided into ten subtypes, adenocarcinoma (ADC) is the most prevalent one, with the most common predominant type—acinar pattern [8]. Small cell lung cancer (SCLC) is distinguished from NSCLC by its rapid doubling time, high growth fraction, and the early development of widespread metastases [9]. Lungs are also a frequent target for metastases of extrapulmonary cancers with or without known primary tumors. Metastases of extrapulmonary tumors are found in 20 to 50% of cases and 4% of them are represented by metastatic carcinoma of unknown primary localization [10]. GC is the fourth most common malignancy and is still the second cause of death by malignancy worldwide [11]. The disease becomes symptomatic in an advanced stage [12]. It usually occurs sporadically and mainly affects people over the age of 45, highlighting the fact that males are two times more often affected than females [13]. From the morphological point of view, according to the World Health Organization guidelines, the most frequent subtype is the tubular adenocarcinoma, followed by the papillary and mucinous types [14]. Based on the Lauren classification, most studies showed the intestinal subtype to be the most common, followed by the diffuse and then the indeterminate type [15, 16]. Hematogenous metastases to the stomach are rare, despite its rich blood supply, with a postmortem incidence of 0.2–9% [17]. Metastatic lung cancer to the gastrointestinal (GI) tract is uncommon and very rare, ranging from 0.5 to 14% of cases [17]. They are commonly asymptomatic, and the diagnosis is usually proved during autopsy.

A differential diagnosis between primary lung adenocarcinoma and pulmonary metastasis from another primary site is essential for setting up of a proper treatment. Ancillary tests are extremely helpful, especially when the biopsy results are histologically ambiguous, the most useful biomarkers being Napsin A and thyroid transcription factor-1 (TTF-1) [18, 19].

The main purpose of this study is to highlight the importance of morphological and immunohistochemical tests to distinguish the origin of the primary tumor, in the case of a 65-year-old deceased male, presenting multiple tumors within the lung, stomach, kidneys and adrenal organs.

## 2. Case Presentation

### 2.1. Clinical Findings

**Figure 1:**
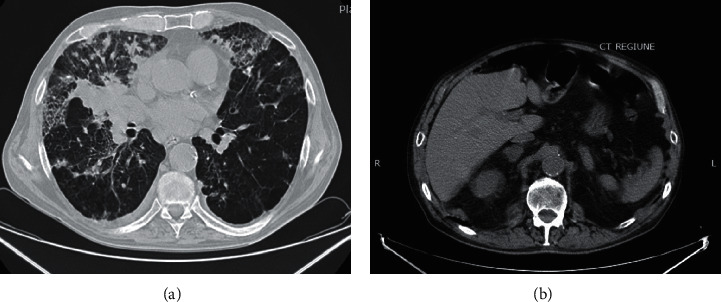
Thoracic CT at the initial diagnosis, without intravenous administration of contract material (increased creatinine levels): (a) dense mediastinal pulmonary mass localized in the medium lobe of the right lung, which meets the pulmonary hilum, and cannot be separated from the mediastinal structures, mediastinal, and retrocrural lymphadenopathies; (b) adrenal glands with diffuse increased sizes and spontaneous tissue density.

We report a case of multiple tumors found in the pulmonary, gastric, renal, and adrenal territories, in a 65-year-old overweight and chronic smoking male patient. His medical history revealed a right pulmonary tumor with adrenal metastases, assessed at the Clinical Pneumophtisiology Hospital of Constanta, Romania, in November 2019. In December 2019, he presented to the Emergency Department of “St. Andrew” County Hospital, Constanta, Romania, complaining of dyspnea with a range of 77% in oxygen saturation, fatigability, and productive cough. The computed tomography (CT) scan of the chest showed minimal right pleural effusion with an ipsilateral pulmonary mass, lymphadenopathy of the mediastinal and retrocrural spaces, and bilateral adrenal masses (Figures 1(a) and 1(b)). The laboratory tests showed neutrophilic leukocytosis, a nonspecific inflammatory syndrome, severe azotic retention with low levels of creatinine clearance, and an estimated glomerular filtration rate suggestive of chronic renal failure. During the hospitalization, the patient was treated with antibiotics, diuretics, systemic corticosteroids, and expectorants, followed by amelioration of the respiratory symptoms. Unfortunately, the patient's condition quickly deteriorated, he passed away, and an autopsy was performed.

### 2.2. Autopsy Findings

**Figure 2:**
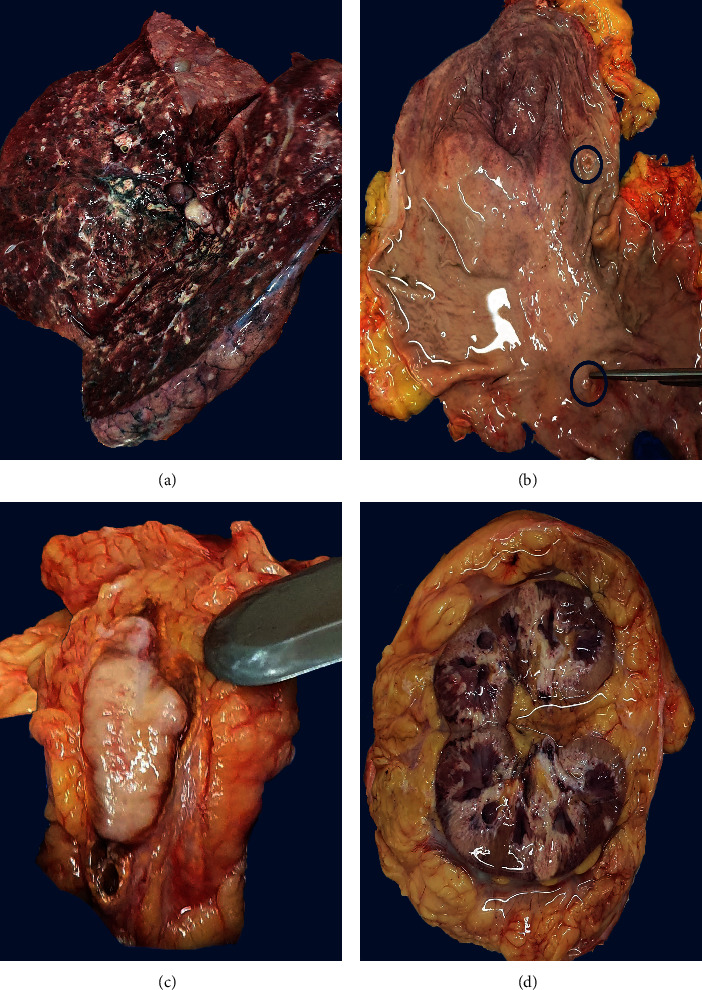
Gross findings of the autopsy specimens: (a) 4 × 3 cm poorly defined lesion in contact with the pulmonary hilum, and multiple foci of necrotic nodules, localized in the right lung. (b) Gross view of the gastric mucosa with one ulcerative, infiltrative lesion. (c) Whitish-yellow, poorly defined lesion in the right adrenal gland. (d) Left kidney showing a surface of cut section with multiple poorly defined lesions with diffuse disposition.

The autopsy revealed a male patient weighing 72 kg and measuring 167.0 cm in length (body mass index: 25.82). Macroscopically, a 4 × 3 cm poorly defined lesion was found in the middle lobe of the right lung, with multiple bilateral necrotic nodules, varying in size from 0.5 cm to 1.5 cm in diameter (Figure 2(a)). The gastric examination diagnosed an ulcerative and infiltrative lesion, measuring 1 cm in the greatest dimension (Figure 2(c)). Moreover, multiple poorly defined lesions were found in both kidneys and the adrenal glands (Figures 2(b) and 2(d)). The specimens obtained from the autopsy were sent to the Clinical Service of Pathology for morphological evaluation.

#### 2.2.1. Microscopical Features

**Figure 3:**
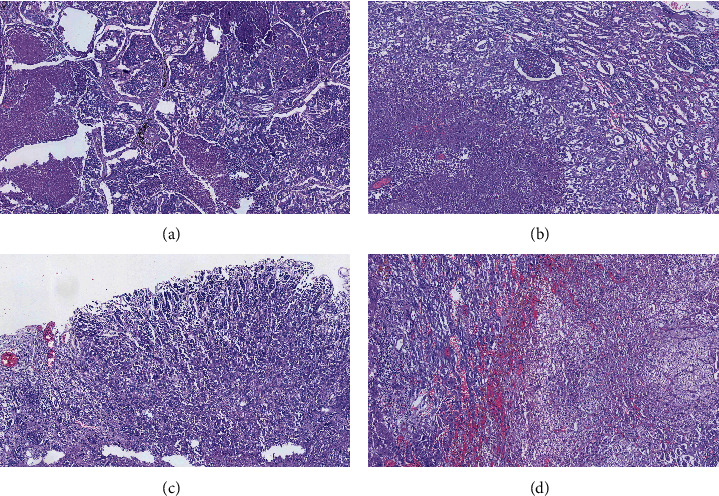
Microscopical features of (a) lung tumor composed of malignant cells, predominantly arranged in an alveolar growth pattern, defined by solid sheets and tumor nests (H&E, 40X); (c) gastric mucosal lesion-malignant epithelial proliferation with adenocarcinoma morphology (H&E, 100X); (b) renal lesion; and (d) adrenal lesion-malignant epithelial proliferation with cytological and architectural modifications like that found in the lungs, presenting with massive alveolar growth pattern and extensive foci of necrosis (H&E, 40X).

The selected autopsy specimens were fixed in 10% formalin and paraffin-embedded, and then stained with hematoxylin and eosin (H&E). The histopathological examination of the standard pulmonary samples revealed a malignant polygonal epithelial proliferation, with pleomorphic nuclei, frequent atypical mitoses, with a clear nucleoli, and moderately eosinophilic cytoplasm. Malignant cells were predominantly arranged in a solid growth pattern, focally presenting papillary features (Figure 3(a)). Lymphovascular invasion and tumoral necrosis were also noted. The presumptive morphological diagnosis was primary lung adenocarcinoma.

Renal and adrenal lesions were characterized by malignant epithelial proliferation with cytological and architectural modifications like those found in the lungs, presenting with a massive alveolar growth pattern, extensive foci of necrosis, and neoplastic cell emboli in the lymphovascular spaces (Figures 3(c) and 3(d)).

Morphological evaluation of the lesion found in the stomach also revealed a malignant epithelial proliferation of the gastric mucosa with glandular differentiation, which extended to the muscular layer, accompanied by lymphovascular invasion (Figure 3(b)). The cytological and architectural features were consistent with the diagnosis of a moderately differentiated primary gastric adenocarcinoma. These findings raised the question whether the lung tumor was a primary mass, or a metastasis from the malignant tumor of the stomach.

The slides were evaluated by a Nikon Eclipse E600 microscope and representative photos were taken from digital whole slide images, obtained with a HuronTISSUEScope^TM^ 4000XT scanner [20].

### 2.3. Immunohistochemistry Evaluation

**Figure 4:**
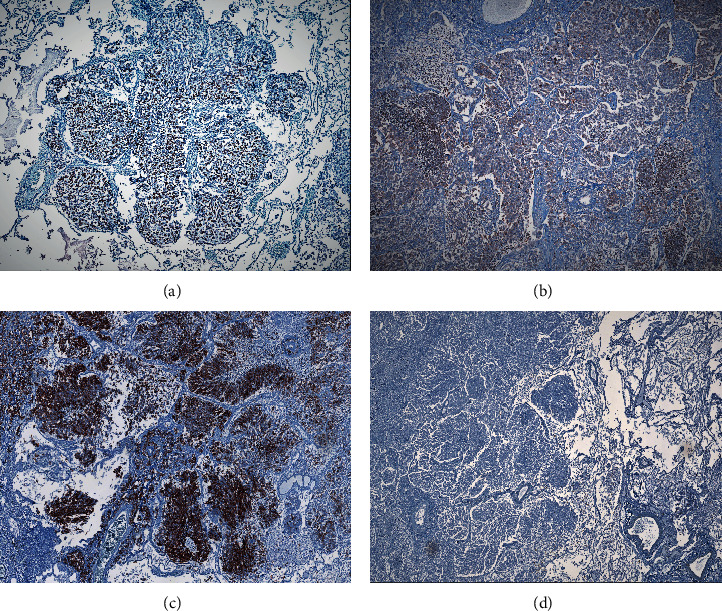
Immunohistochemistry evaluation of the lung surgical specimen. (a) 90% positive nuclear immunostaining for TTF-1 biomarker (IHC; 40X). (b) 85% positive cytoplasmic immunostaining for Napsin A biomarker (IHC; 40X). (c) Diffuse and intense membranous staining reaction for CK7 biomarker (IHC; 40X). (d) Negative immunostaining for CK20 biomarker (IHC; 40X).

Immunohistochemical evaluation was performed on four-*μ*m thick sections of a representative formalin-fixed, paraffin embedded tissue block from the lung tumor. After epitope retrieval, tissue sections were incubated with a panel of seven monoclonal mouse antibodies, ready to use, from BIOCARE Medical (Table 1). A positive reaction for the TTF-1 biomarker was seen considering that at least 1% of the tumor cells were positive [21] and for Napsin A, when a cytoplasmic staining of more than 10% of the tumor cells was found [22]. In the present study, a positive immunoprofile for TTF-1 (Figure 4(a)), a cytoplasmic immunostaining for Napsin A (Figure 4(b)) and a diffuse and intense membranous staining reaction for the cytokeratin 7 (CK7) biomarker (Figure 4(c)) were remarked. Negative results were noticed for cytokeratin 20 (CK20), p40, synaptophysin, and chromogranin A biomarkers (Figure 4(d)).

## 3. Discussion

MPMNS are very rare tumors which pose difficulties in setting up a correct diagnosis, in conjunction with planning a prompt therapeutic protocol. According to Warren and Gates criteria, the diagnosis of MPMN requires the following mandatory criteria: (a) each tumor should present a definite picture of malignancy, (b) each tumor should be histologically distinct, and (c) the possibility that one is the metastasis of the other must be excluded [2]. Clinically, MPMN is often confused with the metastasis or recurrence of primary malignant tumors since both are characterized by new lesions [23]. Metastatic tumors derive from primary lesions, with the same pathological characteristics and similar developmental processes and prognosis [24]. Conversely, MPMN refers to the development of a *de novo* malignant lesion, with morphological features completely different from those of the original tumor, consequently involving a different prognosis [24, 25].

Globally, earlier studies showed that the combination of gastric and lung cancers is very rare, with a maximum 0.4% incidence [26, 27]. In the study of Duchateau et al., only three patients from 860 NSCLC patients had synchronous or metachronous GC [27]. In the present report, we add new insights to this simultaneous occurrence of two different tumors and highlight the role of ancillary tests in their confirmation.

According to a retrospective study of Kurishima et al., the proportion of men was higher among patients with lung cancer and GC than those without, while the proportion of smokers was higher among lung cancer patients with GC than those without [5]. About twenty-seven of the 45 patients had smoking-related cell types of lung cancer (squamous cell carcinoma and small-cell lung cancer) and the proportion of these morphological subtypes was elevated in patients with lung cancer and GC than those who did not present both lung cancer and GC [5]. In the present report, we noticed that the patient was a smoker for more than 20 years, considered a major risk factor for LC associated with GC.

GC is the second most common cancer worldwide and stands for 8% of all cancers, causing more deaths than lung cancer [28]. Despite the decreasing worldwide incidence, GC accounts for 3% to 10% of all cancer-related deaths [29]. Gastric metastasis from lung adenocarcinoma is extremely rare and this process is not yet completely explained. One hypothesis suggests that certain cytokines may affect the organ specificity in hematogenous metastases, which are usually submucosal and sometimes ulcerated [17]. These lesions are found more commonly in the upper and middle thirds of the stomach, radiographically mirroring a bull's eye sign and endoscopically resembling a volcano or presenting an umbilicated tip [17]. Most of gastric metastases are submucosal; thus, they generally remain asymptomatic [17]. On the other hand, lung cancer is primarily known to be associated with upper aerodigestive cancer [30–33] and lung cancer seems to be one of the most common second primary cancers in patients with GC [30, 31].

In this current case, the morphological features of the tumors found in the lung suggest the diagnosis of solid and papillary adenocarcinoma with secondary metastases in the kidney and adrenal glands. Aiming for a correct diagnosis and excluding differential diagnoses, ancillary tests were applied. Adenocarcinoma (ADC) is the most frequent cell type of lung cancer, accounting for over 50% of cancers [34]. Solid predominant ADC was significantly correlated with male gender and smoking history [35]. It was significantly associated with aggressive tumor characteristics including larger tumor size, higher prevalence of lymphovascular invasion, and lymph node metastasis [35]. In the present case, extensive areas of solid pattern malignant proliferation associated with necrosis, lymphovascular invasion, and lymph node metastasis explained its aggressive behavior.

Immunohistochemical techniques are mandatory for a proper diagnosis. The TTF-1 biomarker is a member of the homeobox protein NKX2 (which in humans is encoded by the NKX2-1 gene) family of homeodomain transcription factors which is found in epithelial cells of the thyroid gland and lung, being expressed in pulmonary ADCs and thyroid carcinomas. It is not found in ADCs arising from other sites and, accordingly, are now routinely used to distinguish primary lung cancer from lung metastasis [36]. TTF-1 staining might be also useful in localizing the tumor origin of ADCs encountered outside of the lung [36]. This biomarker has been shown to stain approximately 65–70% of lung ADC cases and rarely provokes a positive reaction in lung squamous cell carcinomas (SCC) [37–39]. Napsin A is an aspartic proteinase expressed in type II pneumocytes, highly sensitive, specific marker for lung ADC, as 85–90% of patients have a positive immunostain, while a negative reaction has been seen in nearly all cases of lung SCC [37–39]. The antibody cocktail of TTF-1 + Napsin A has proved a sensitivity of 91% and 95% specificity for lung ADC versus lung SCC [37]. In cases where TTF-1 and Napsin A are both positive, the specificity of lung ADC versus lung SCC has been shown to be 100% [37]. Considering the present case of MPMN, the positive antibody cocktail of TTF-1 + Napsin A confirms earlier studies of high sensitivity and specificity for lung ADC.

Metastatic carcinoma of unknown primary origin is a perplexing but common problem, accounting for up to 10% to 15% of all solid tumors at presentation [36]. TTF-1 is expressed in lung ADCs and thyroid carcinomas but not in ADCs arising from other sites [36], which confirms the results in this case of primary lung ADC.

The immunohistochemical expression patterns of CK7 and CK20 biomarkers constitute a useful tool to distinguish the site of origin of metastatic carcinomas when a malignant gastrointestinal tumor is simultaneously found. According to the survey conducted by Chu et al. on 435 cases of epithelial neoplasms, the vast majority of cases of ADCs was positive for CK7, including lung (100%) and—in contrast—only a small percentage of cases of gastric ADC (28%) were CK7-positive [40]. Based on the differential pattern of CK staining reported in specialized literature, we decided to currently evaluate the CK expression in tissue samples of lung adenocarcinoma. In comparison with the mentioned studies, in the present case, the characteristic features of a positive stain for TTF-1 and Napsin A found in the lung tumor and the CK7+/CK20− confirmed the diagnosis of primary lung ADC and invalidated the hypothesis of a metastasis of gastric adenocarcinoma or another forms of lungs cancer.

Based on these results, the final diagnosis was synchronous MPMN composed of non-small-cell lung ADC, presenting a solid growth pattern, with secondary determination in the kidney and adrenal gland, and low grade/moderately differentiated gastric adenocarcinoma.

Our results suggest that patients with lung cancer should be counselled at the time of diagnosis about their increased risk of developing second cancers, including GC.

## 4. Conclusions

This is a case report of synchronous lung and gastric ADCs, with an aggressive evolution, in a male patient with smoking history. The gastric lesions were diagnosed only at autopsy and the lung tumors showed a solid growth pattern and were positive for TTF-1, Napsin A, CK7, and negative for CK20.

The increased incidence of multiple malignant tumors is a challenge for physicians; an early diagnosis is essential for the patient to be eligible for radical treatment, which thereafter shows an improved prognosis. Thorough medical monitorization of elderly males present or ex-smokers with lung cancer is recommended.

We also highlight the importance of the ancillary test to distinguish a primary tumor from a metastatic one because the diagnosis of the metastatic carcinoma of unknown origin can be very difficult and the determination of the primary site of the metastasis is a challenge to both oncologists and pathologists, potentially having important clinical and therapeutic consequences.

## Figures and Tables

**Table 1:** Antibodies used for immunohistochemical evaluation.

Antibody	Isotype	Clone	Antigen retrieval	Dilution	External control
Thyroid transcription factor-1 (TTF-1)	IgG1	8G7G3/1	TTF-1	1 : 100	Lung adenocarcinoma
Napsin A	IgG1	TMU-Ad 02	Synthetic peptide of a part of the N-terminus of human Napsin A	1 : 100	Lung adenocarcinoma
CK7	IgG1	OV-TL 12/30	CK7	1 : 100	Ovarian or breast cancer
CK20	IgG2a	Ks20.8	CK20	1 : 100	Colon carcinoma
p40(M)	IgG1	BC28	Amino acids 5–17 of p40	1 : 100	Lung squamous cell carcinoma
Synaptophysin	IgG1	27G12	Synaptophysin	1 : 150	Small cell lung carcinoma
Chromogranin A	IgG1 + IgG1	LK2H10 + PHE5	Chromogranin A	1 : 100	Pancreas or adrenal gland

## Data Availability

The data used for the findings of this article are available and will be provided by the corresponding author upon request.

## References

[1] Liu Z., Liu C., Guo W., Li S., Bai O. (2015). Clinical analysis of 152 cases of multiple primary malignant tumors in 15,398 patients with malignant tumors. *PLoS One*.

[2] Warren S., Gates O. (1932). A survey of the literature and statistical study. *American Journal of Cancer*.

[3] Chirila D. N., Turdeanu N. A., Constantea N. A. (2013). Multiple malignant tumors. *Chirurgia*.

[4] (1998). SEER program code manual. https://seer.cancer.gov/tools/codingmanuals/historical.html.

[5] Kurishima K., Satoh H., Kagohashi K. (2009). Patients with lung cancer with metachronous or synchronous gastric cancer. *Clinical Lung Cancer*.

[6] Hou S., Zhou S., Qin Z. (2017). Evidence, mechanism, and clinical relevance of the transdifferentiation from lung adenocarcinoma to squamous cell carcinoma. *American Journal of Pathology*.

[7] Goldblum J. R., Zander D. S., Farver C. F. (2018). *Pulmonary Pathology: A Volume in the Series Foundations in Diagnostic Pathology*.

[8] Travis W. D., Brambilla E., Burke A. P., Marx A., Nicholson A. G. (2015). *WHO Classification of Tumours of the Lung, Pleura, Thymus and Heart*.

[9] Byers L. A., Michael Gay C. (2019). *Pathobiology and Staging of Small Cell Carcinoma of the Lung*.

[10] Fisseler-Eckhoff A., Müller K. M. (2000). Differential diagnosis of primary lung tumors and pulmonary metastases. *Verhandlungen der Deutschen Gesellschaft für Pathologie*.

[11] Jemal A., Bray F., Center M. M., Ferlay J., Ward E., Forman D. (2011). Global cancer statistics. *CA: A Cancer Journal for Clinicians*.

[12] Sitarz R., Skierucha M., Mielko J., Offerhaus J., Maciejewski R., Polkowski W. P. (2018). Gastric cancer: epidemiology, prevention, classification, and treatment. *Cancer Management and Research*.

[13] Forman D., Burley V. J. (2006). Gastric cancer: global pattern of the disease and an overview of environmental risk factors. *Best Practice and Research Clinical Gastroenterology*.

[14] Lokuhetty D., White V. A., Watanabe R. (2019). *WHO Classification of Tumours-Digestive System Tumours*.

[15] Berlth F., Bollschweiler E., Drebber U., Hoelscher A. H., Moenig S. (2014). Pathohistological classification systems in gastric cancer: diagnostic relevance and prognostic value. *World Journal of Gastroenterology*.

[16] Shi C., Berlin J., Branton P. A. (2020). *Protocol for the Examination of Specimens from Patients with Carcinoma of the Stomach*.

[17] Qasrawi A., Ghanimeh M. A., Albadarin S., Yousef O. (2017). Gastric metastases from lung adenocarcinoma causing gastrointestinal bleeding. *ACG Case Reports Journal*.

[18] Kim M. J., Hong J. H., Park E. S., Byun J. H. (2015). Gastric metastasis from primary lung adenocarcinoma mimicking primary gastric cancer. *World Journal of Gastrointestinal Oncology*.

[19] Cozaru G. C., Aschie M., Mitroi A. F., Bălțătescu G. I., Nicolau A. A., Poinăreanu I. (2014). Correlation between Her2 expression and clinic-pathological features of invasive gastric carcinoma. *The 26th European Congress of Pathology*.

[20] Baltatescu G. I., Aschie M., Deacu M. (2020). The role of artificial intelligence for image analysis in surgical pathology. *Economics and Applied Informatics*.

[21] Vidarsdottir H., Tran L., Nodin B. (2018). Comparison of three different TTF-1 clones in resected primary lung cancer and epithelial pulmonary metastases. *American Journal of Clinical Pathology*.

[22] Yamashita Y., Nagasaka T., Naiki-Ito A. (2015). Napsin A is a specific marker for ovarian clear cell adenocarcinoma. *Modern Pathology*.

[23] Cigna E., Gradilone A., Sorvillo V., Scuderi N. (2011). ABCB5 in peripheral blood of a patient affected by multiple primary malignancies. *Annali Italiani di Chirurgia*.

[24] Xu L. L., Gu K. S. (2014). Clinical retrospective analysis of cases with multiple primary malignant neoplasms. *Genetics and Molecular Research*.

[25] Papajík T., Mysliveček M., Sedová Z. (2011). Synchronous second primary neoplasms detected by initial staging F-18 FDG PET/CT examination in patients with non-hodgkin lymphoma. *Clinical Nuclear Medicine*.

[26] Eom B. W., Lee H. J., Yoo M. W. (2008). Synchronous and metachronous cancers in patients with gastric cancer. *Journal of Surgical Oncology*.

[27] Duchateau C. S., Stokkel M. P. (2005). Second primary tumors involving non-small cell lung cancer: prevalence and its influence on survival. *Chest*.

[28] Weisenberg E. (2020). Carcinoma-general. Pathology outlines.com website. https://www.pathologyoutlines.com/topic/stomachcarcinomageneral.html.

[29] Crawford J. (1994). *The Gastrointestinal Tract. Pathologic Basis of Disease*.

[30] Ikeguchi M., Ohfuji S., Oka A., Tsujitani S., Maeda M., Kaibara N. (1995). Synchronous and metachronous primary malignancies in organs other than the stomach in patients with early gastric cancer. *Hepato-Gastroenterology*.

[31] Ikeda Y., Saku M., Kawanaka H., Nonaka M., Yoshida K. (2003). Features of second primary cancer in patients with gastric cancer. *Oncology*.

[32] Ishida T., Saitoh G., Maruyama R. (1995). Survival following resection for lung cancer as a second primary cancer. *International Surgery*.

[33] Kamiyama H., Ikeya T., Suda K., Murai K., Aoyama K., Hoshi E. (2004). Second primary digestive cancer after resection of lung cancer. *Surgery Today*.

[34] Zander D. S., Farver C. F. (2018). *Pulmonary Pathology - Second Edition - a Volume in the Series Foundations in Diagnostic Pathology*.

[35] Zhang Y., Li J., Wang R. (2014). The prognostic and predictive value of solid subtype in invasive lung adenocarcinoma. *Scientific Reports*.

[36] Srodon M., Westra W. H. (2002). Immunohistochemical staining for thyroid transcription factor-1: a helpful aid in discerning primary site of tumor origin in patients with brain metastases. *Human Pathology*.

[37] Tacha D., Yu C., Bremer R., Qi W., Haas T. (2012). A 6-antibody panel for the classification of lung adenocarcinoma versus squamous cell carcinoma. *Applied Immunohistochemistry and Molecular Morphology*.

[38] Mukhopadhyay S., Katzenstein A. L. A. (2011). Subclassification of non-small cell lung carcinomas lacking morphologic differentiation on biopsy specimens: utility of an immunohistochemical panel containing TTF-1, napsin A, p63, and CK5/6. *The American Journal of Surgical Pathology*.

[39] Turner B. M., Cagle P. T., Sainz I. M., Fukuoka J., Shen S. S., Jagirdar J. (2012). Napsin A, a new marker for lung adenocarcinoma, is complementary and more sensitive and specific than thyroid transcription factor 1 in the differential diagnosis of primary pulmonary carcinoma: evaluation of 1674 cases by tissue microarray. *Archives of Pathology and Laboratory Medicine*.

[40] Chu P., Wu E., Weiss L. M. (2000). Cytokeratin 7 and Cytokeratin 20 expression in epithelial neoplasms: a survey of 435 cases. *Modern Pathology*.

